# 1437. Investigation of Increased Latent Tuberculosis Infection Rates During the COVID-19 Pandemic Among International Students at a U.S. University Campus

**DOI:** 10.1093/ofid/ofac492.1266

**Published:** 2022-12-15

**Authors:** Wajih Askar, Lauri E Nandyal, RaeJean Hardig, Tina Coberley, Amanda Doner, Lisa Haglund, Jennifer W Forrester, Shu-Hua Wang, Michael B Holliday, Moises A Huaman

**Affiliations:** University of Cincinnati, Cincinnati, Ohio; University of Cincinnati, Cincinnati, Ohio; University of Cincinnati, Cincinnati, Ohio; University of Cincinnati, Cincinnati, Ohio; University of Cincinnati, Cincinnati, Ohio; Division of Infectious Diseases, University of Cincinnati, Cincinnati, Ohio; University of Cincinnati, Cincinnati, Ohio; The Ohio State University, Columbus, Ohio; University of Cincinnati, Cincinnati, Ohio; Division of Infectious Diseases, University of Cincinnati, Cincinnati, USA, Cincinnati, OH

## Abstract

**Background:**

The COVID-19 pandemic has caused dramatic changes in the epidemiology of many diseases globally due to various changes in exposure to different pathogens, social restrictions, and demographic shifts. A university student health center located in the U.S. Midwest detected an increase in latent tuberculosis infection (LTBI) rate among its incoming international students (INTS) from 5.7% to 8.1% in the fall semesters of 2019 and 2021, respectively. We describe our approach to investigating the increase in LTBI rate at a university campus in a low-endemicity area.

**Methods:**

Factors that may affect LTBI rates were evaluated. LTBI testing policy and methods were reviewed. Medical and lab staff were interviewed regarding the consistency of specimen collection, transport, and processing. LTBI risks in the general population such as older age, male gender, and country of origin (COO) were also considered. Factors that were expected to be uncommon in the INTS (homelessness, incarceration, and illicit drug abuse) were not evaluated.

**Results:**

No changes in the INTS screening policy were noted. All incoming INTS were screened for LTBI during initial health screening, regardless of COO. The same manufacturer's QuantiFERON®-TB Plus test was utilized. Compared to previous years, no inconsistencies in the testing logistics were reported. A total of 1,016 INTS were screened in 2019 and 1,179 in 2021. There were no significant differences in average age in years (23.1 vs. 23.3) or male gender (59.6% vs. 56.8%) between 2019 and 2021, respectively. Most INTS came from two countries (A and B). Country A was COO of 21.6% of INTS in 2019, which dropped to 8.4% in 2021. Country B was COO of 44.8% of INTS in 2019, which increased to 57.6% in 2021 (*p*< 0.001; Figure 1). Although LTBI rates within each country (A and B) remained similar before and during the COVID-19 pandemic (Figure 2), country B had consistently higher rates than country A (*p*< 0.001), which contributed to the overall increased rate of LTBI in 2021.

**Figure 1.**
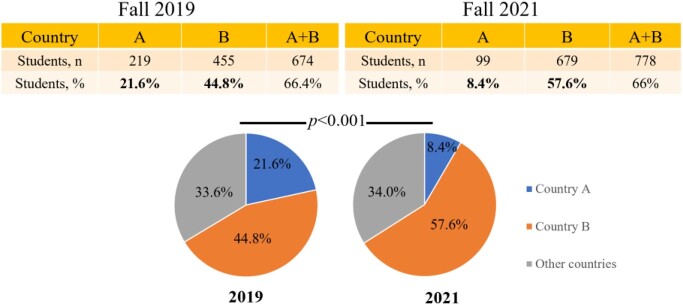
Numbers and percentages of international students from countries A and B.

**Figure 2.**
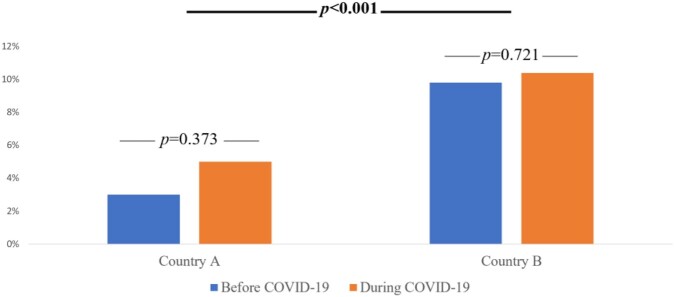
LTBI positivity rates for countries A and B before and during COVID-19 pandemic.

**Conclusion:**

Evaluating changes in COO of INTS is essential in investigating trends in LTBI rates at otherwise low-endemicity universities. In our investigation, demographic changes in university admissions over two years relative to COVID-19 pandemic restrictions contributed to an increase in the overall LTBI rate.

**Disclosures:**

**Moises A. Huaman, MD, MSc**, Gilead: Grant/Research Support|Insmed: Grant/Research Support.

